# Temporal Release and Denature of Several Mediators in Pure Platelet-Rich Plasma and Temperature-Induced Platelet Lysates Derived from a Similar Bovine Platelet Concentrate

**DOI:** 10.1155/2022/2609508

**Published:** 2022-09-23

**Authors:** Jorge U. Carmona, Catalina López, Alejandro Ceballos-Márquez

**Affiliations:** ^1^Grupo de Investigación Terapia Regenerativa, Departamento de Salud Animal, Universidad de Caldas, Manizales, Colombia; ^2^Grupo de Investigación Patología Clínica Veterinaria, Departamento de Salud Animal, Universidad de Caldas, Manizales, Colombia; ^3^Grupo de Investigación Calidad de La Leche y Epidemiología Veterinaria (CLEV), Departamento de Producción Agropecuaria, Universidad de Caldas, Manizales, Colombia

## Abstract

There is scarce information about bovine platelet-rich plasma/platelet-rich gel (PRP/PRG) and related hemocomponents (HCs), such as platelet lysates (PLs), including growth factor (GF) and cytokine concentrations, and how the stability of these biomolecules could be affected by time and temperature. This study aimed to evaluate the release and stability of transforming growth factor beta 1 (TGF-*β*_1_), interleukin 4 (IL-4), and tumor necrosis factor alpha (TNF-*α*) contained in bovine pure PRP (P-PRP) and temperature-induced PL (TIPL) coming from a similar platelet concentrate (PC) at 4 and 37°C at 3 and 96 h. Platelet concentrates (PCs) presented a 1.7-fold concentration of platelets (PLTs) with negligible counts of white blood cells (WBCs) when compared to the counts for these cells in whole blood. TGF-*β*_1_ concentrations were significantly lowest in plasma followed by TIPL, chemical-induced PL (CIPL), and P-PRP. IL-4 and TNF-*α* concentrations did not differ between HCs. TGF-*β*_1_ concentrations were negatively affected in P-PRPs stored at 4°C at 3 and 96 h, whereas those from P-PRP maintained at 37°C presented similar concentrations to TIPL stored at both temperatures over time. IL-4 and TNF-*α* concentrations were not affected by time or temperature in any of the HCs evaluated. Pure PRGs released additional quantities of GF and cytokines over time when compared with HCs stored over 96 h at 4 and 37°C. The method, either chemical or physical, used for platelet activation or damage produces a different GF and cytokine release pattern, which makes to each evaluated HCs different despite they come from a similar bovine PC. P-PRP activated with calcium gluconate and maintained at 37°C, which polymerizes in P-PRG, showed the best GF and cytokine release/denature profile compared with the rest of the HCs evaluated.

## 1. Introduction

Platelets (PLTs) are considered to have a complex role in wound healing because they not only mechanically prevent bleeding in injured tissues but also secrete granular contents rich in polypeptides (i.e., cytokines and growth factors (GFs)) with anti-inflammatory and anabolic properties over the wound healing process [1, 2]. Furthermore, they also release antimicrobial peptides and act as a first-line barrier in contaminated sites produced by trauma [3]. There are a plethora of PLT-related products or hemocomponents (HCs) used in human and veterinary medicine that are sourced from anticoagulated whole blood [4]. These HCs could include platelet-rich plasma (PRP) products and platelet lysates (PLs) [5, 6].

PRP is a hemocomponent for topical or infiltrative use that has an autologous or allogeneic origin. It is obtained from the aggregation of a platelet concentrate mixed with activating substances, such as calcium salts or thrombin [5]. PRP can be classified according to its white blood cell (WBC) concentration in pure PRP (P-PRP), which is a platelet concentrate with a lower or negligible concentration of WBCs, whereas when these last cells are detectable at higher concentrations is named a leukocyte-PRP (L-PRP) [7].

PL is a protein extract obtained from the fractionation of platelet concentrate by physical and chemical means. This hemocomponent is mostly used as a supplement for cell growth in vitro. More rarely, PL is studied and clinically used as a therapeutic agent for topical application [5]. Notably, a PL can be produced by several chemical and physical methods that include, among others, the addition of an anionic detergent [8, 9], the use of extreme temperature changes [8, 10, 11], and sonication [12].

According to the revised literature, there are scarce published studies comparing the GF and cytokine release kinetics in bovine HCs, such as PRP and PL, and how the stability of these biomolecules (mediators) could be affected by factors such as time and temperature. These aspects are of paramount importance when it is planned to produce large volumes of homologous HCs, as happens when it is expected to treat several cows with clinical or subclinical mastitis in dairy farms [11, 13].

The present study aimed to evaluate and compare the temporal release and stability of transforming growth factor beta 1 (TGF-*β*_1_), interleukin 4 (IL-4), and tumor necrosis factor alpha (TNF-*α*) from P-PRP and temperature-induced PL (TIPL) derived from bovine platelet concentrates. We hypothesized that although either P-PRP or TIPL could come from a similar platelet concentrate, their growth factor (GF) and cytokine release/denature profile could be different.

## 2. Materials and Methods

This study was accepted by the Committee for Animal Experimentation of the Universidad de Caldas. The cows included were owned by the farm system of the same institution.

### 2.1. Animals

Whole blood from 6 clinically healthy heifers with a mean age of 20 (±3) months old and with a mean weight of 200 (±20) kg was obtained. Three weeks before the experiment, the animals were housed, dewormed, and underwent physical examination and blood samples were taken for complete blood count and clinical biochemistry to ensure the health status of the heifers.

After sedation with xylazine (0.01 mg/kg, IM) and immobilization of each heifer, 450 mL of whole blood obtained by jugular venipuncture was deposited in a double-transfusion bag with CDPA-1 as an anticoagulant. A 10-mL blood sample was taken from each bag to perform a basal hemogram (Celltac *α* MEK-6450. Nihon Kohden, Tokyo, Japan) and then, plasma was obtained by centrifugation of this sample at 1411*g*/6 min. This last hemocomponent was considered a negative control for GF and cytokine enrichment [9].

In general, the experiment was run by duplicates. Each heifer was evaluated separately during an independent date in relation to the other animals. Thus, we obtained the mean values from all six heifers for each variable evaluated in the study.

### 2.2. Platelet Concentrate Procurement

Transfusion bags containing whole blood were centrifuged at 698*g*/6 min (RotoSilenta 630 RS. Hettich, Tuttlingen, Germany). After this, the plasma from each bag was gently separated and packed in the satellite bag. The hemocomponent deposited in the satellite bag was considered the platelet concentrate. At this point, a sample of this hemocomponent was used for the hemogram, and another sample was used for elaborating a PL with Triton X 100 (PanReacAppliChem, Barcelona, Spain) which was considered a chemical-induced PL (CIPL). This hemocomponent was considered the positive control for GF and cytokine enrichment in platelet concentrate [9].

### 2.3. Pure-Platelet Rich Plasma/Pure-Platelet Rich Gel Procurement

Aliquots of platelet concentrate from each heifer were activated with a 10% calcium gluconate solution (9.3 mg/mL) (Ropsohn Therapeutics Ltda®, Bogotá, Colombia) at a ratio of 9 : 1 to induce PLT activation and, consequently, GF and cytokine release.

### 2.4. Platelet Lysate Procurement

Aliquots of platelet concentrate from each heifer were frozen at −80°C for 30 min and tawed at room temperature for over 30 min for 3 consecutive cycles [8, 10]. This hemocomponent was considered temperature-induced PL (TIPL).

### 2.5. Determination of the Stability of Growth Factors and Cytokines in Hemocomponents at 4 and 37°C over 96 h

Tubes containing either P-PRP or TIPL were maintained in two different temperature conditions (either 4 or 37°C) and sampled at 3 and 96 h to determine the stability of GFs and cytokines over time.

### 2.6. Determination of the Release (Elution) Kinetics of Growth Factors and Cytokines from PRP/PRG at 37°C over 96 h

Clots (platelet-rich gels (PRGs)) from calcium gluconate-activated P-PRP were obtained after a 3 h incubation period and cultured with 4 mL of Dulbecco's modified Eagle's medium (DMEM) (high glucose, 4500 mg/L) with L-glutamine and sodium bicarbonate and free of sodium pyruvate (DMEM, Lonza Group Ltd, Basel, Switzerland) and supplemented with streptomycin (100 *μ*g/mL) and penicillin (100 *μ*g/mL) without the addition of serum. Cultures were incubated in a 5% CO_2_ and water saturated atmosphere. The clots were cultured over 96 h and sampled at 3, 6, 24, 48, and 96 h. Media changes were performed at each time point to estimate the temporal production and release of GF and cytokines over time.

### 2.7. Growth Factor and Cytokine Determination

TGF-*β*_1_, IL-4, and TNF-*α* were measured in hemocomponents and PRG culture media using commercial ELISA development kits. TGF-*β*_1_ (Human TGF-*β*1 DuoSet, DY240E, R&D Systems, Minneapolis, MN, USA) concentrations were determined using human antibodies. Notably, TGF-*β*_1_ shares high homology between humans and cattle [14], and previously, this human GF antibody was used in another bovine PRP study [9]. IL-4 (Mabtech AB, Nacka Strand, Sweden), and TNF-*α* (Bovine TNF-alpha DuoSet ELISA, R & D Systems, Minneapolis, MN, USA) were assayed with bovine-specific antibodies.

**Figure 1:**
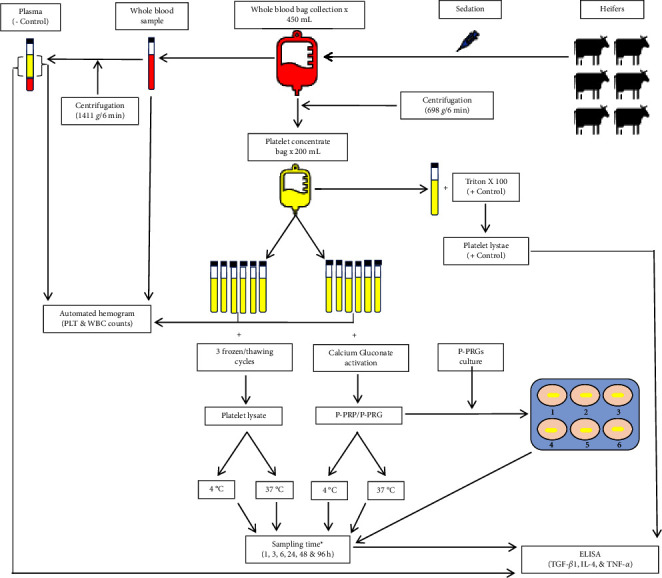
Study's design. IL-4, interleukin 4; PLT, platelet; P-PRP, pure platelet-rich plasma; P-PRG, pure platelet-rich gel; WBC, white blood cell; TGF-b_1_, transforming growth factor beta 1; TNF-*α*, tumor necrosis factor alpha. ^*∗*^P-PRP and platelet lysates induced by temperature were sampled at 3 and 96 h.

The standards provided for each ELISA kit were used to prepare each standard curve according to the manufacture instructions. Readings were performed at 450 nm. In general, the inter- and intra-assay coefficients of variation for the various ELISA kits were between 2 and 5%.  Figure 1 summarizes the study design.

### 2.8. Statistical Analysis

Platelet and WBC counts in whole blood, platelet concentrates, and plasma were compared with one-way ANOVA followed by a Games–Howell test. The concentrations of GF and cytokines in hemocomponents were analyzed by a general linear model (GLM) of repeated measures followed, when necessary, by a Tukey test. A *P* < 0.05 was accepted as statistically significant for all tests.

## 3. Results

### 3.1. Cell Counts in Whole Blood, Platelet Concentrates, and Plasma

**Figure 2:**
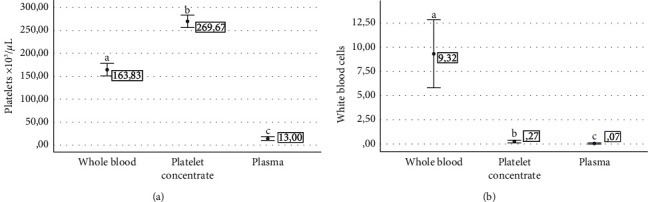
Mean ± sd cell counts in whole blood and hemocomponents. (a) Platelet counts. (b) White blood cell counts. ^a–c^Different lowercase letters denote significant (*P* < 0.001) differences between whole blood and hemocomponents by the Games–Howell post hoc test.

Platelet and WBC counts were significantly (*P* < 0.001) different between whole blood, platelet concentrates, and plasma, with the highest PLT counts for platelet concentrates, followed by whole blood and plasma (Figure 2(a)), whereas WBC counts were highest for whole blood, followed by platelet concentrates and plasma (Figure 2(b)).

### 3.2. Basal Concentrations of Growth Factors and Cytokines in Hemocomponents at 0 h

TGF-*β*_1_ concentrations were significantly different between plasma, and hemocomponents with the lowest concentration for plasma followed by TIPL, chemical-induced PL (CIPL), and P-PRP.

**Figure 3:**
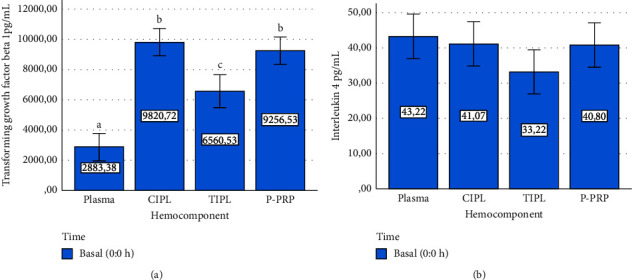
Mean ± sd basal mediator concentration in hemocomponents. (a) Transforming growth factor beta 1. (b) Interleukin 4. ^a–c^Different lowercase letters denote significant (*P* < 0.001) differences between the hemocomponents by the Games–Howell post hoc test.

**Figure 4:**
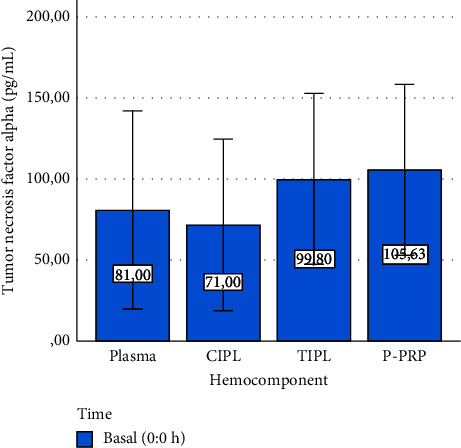
Mean ± sd basal tumor necrosis factor alpha concentration in hemocomponents.

However, the concentrations for this GF in CIPL and P-PRP were similar (Figure 3(a)), whereas IL-4 (Figure 3(b)) and TNF-*α* concentrations did not differ between hemocomponents (Figure 4).

### 3.3. Stability of Growth Factor and Cytokines in Hemocomponents at 4 and 37°C at 3 and 96 h

Hemocomponents (P-PRP or TIPL) stored at both temperatures presented significantly different TGF-*β*_1_ concentrations at 3 h, with the highest values for TIPLs in comparison to P-PRPs. In particular, those TIPLs maintained at 4°C exhibited the most significantly high.

**Figure 5:**
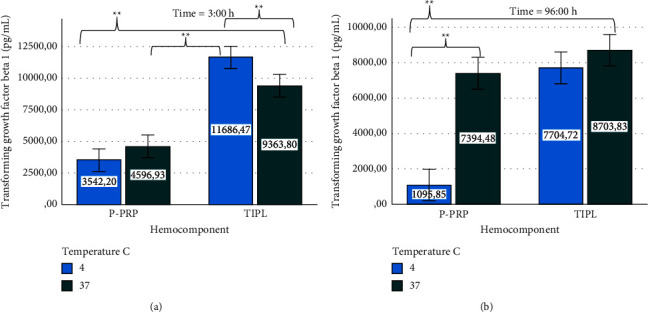
Mean ± sd transforming growth factor beta 1 concentration in hemocomponents affected by time and temperature. (a) Transforming growth factor beta 1 at 3 h. (b) Transforming growth factor beta 1 at 96 h^*∗∗*^ denotes significant (*P* < 0.005) differences for both hemocomponents by the Games–Howell post-hoc test.

GF concentration in comparison with all hemocomponents evaluated at different temperatures (Figure 5(a)). However, at 96 h, P-PRPs stored at 4°C presented significantly lower concentrations than the same hemocomponent stored at 37°C and TIPLs maintained at both temperatures. Notably, at 96 h, TGF-*β*_1_ concentrations from P-PRP maintained at 37°C presented GF concentrations similar to those observed in TIPL stored at both temperatures (Figure 5(b)).

**Figure 6:**
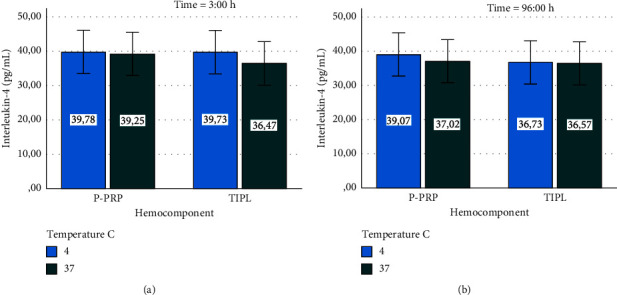
Mean ± sd interleukin-4 concentration in hemocomponents affected by time and temperature. (a) Interleukin-4 at 3 h. (b) Interleukin-4 at 96 h.

IL-4 concentrations remained stable in both hemocomponents stored at 4 and 37°C for 3 and 96 h (Figures 6(a) and 6(b)). In general, the concentrations of this anti-inflammatory cytokine remained similar between the hemocomponents evaluated, including those at time 0 h (Figure 3(b)).

**Figure 7:**
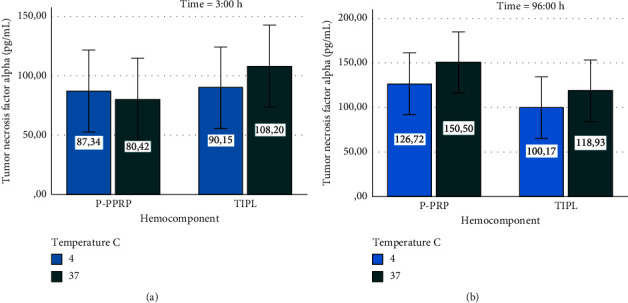
Mean ± sd tumor necrosis factor alpha concentration in hemocomponents affected by time and temperature. (a) Tumor necrosis factor alpha at 3 h. (b) Tumor necrosis factor alpha at 96 h.

TNF-*α* concentrations exhibited a stable concentration pattern in P-PRP and TIPL stored at both temperatures over 3 and 96 h (Figures 7(a) and 7(b)). In general, the concentrations of TNF-*α* remained similar between the hemocomponents evaluated, including those from time 0 h (Figure 4).

### 3.4. Release (Elution) Kinetics of Growth Factor and Cytokines from P-PRP/PRG over 96 h

**Figure 8:**
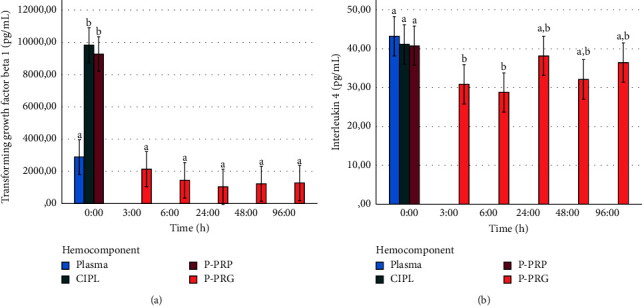
Mean ± sd temporal release of transforming growth factor b 1 (a) and interleukin 4 (b) concentrations from P-PRGs to culture media. ^a–c^Different lowercase letters denote significant (*P* < 0.001) differences over the time by the Games–Howell post hoc test.

The addition of calcium gluconate to platelets concentrates unchained P-PRP release over the first hour and clot formation (P-PRG) at 3 h. At this point, P-PRG was maintained in Culture conditions over 96 h with complete media changes at 6, 24, and 48 h.  Figure 8(a) shows the temporal release of TGF-*β*1 from P-PRGs to culture media. In general, the concentrations released for this GF were significantly (*P* < 0.001) lower at all times when compared to P-PRP and CIPL at time 0 h.

**Figure 9:**
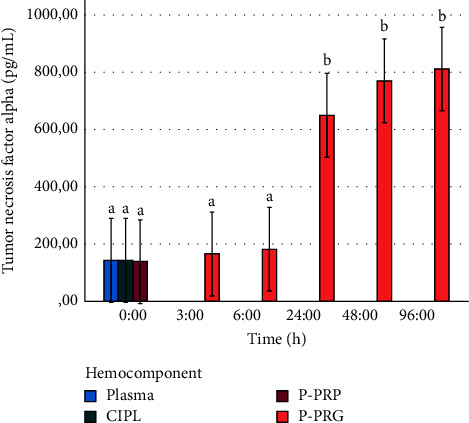
Mean ± SD temporal release of tumor necrosis factor alpha concentrations from P-PRGs to culture media. ^a–c^Different lowercase letters denote significant (*P* < 0.001) differences over the time by the Games–Howell post hoc test.

Temporal IL-4 release was maintained at all time points with a slight release increase at 24, 48, and 96 h (Figure 8(b)), whereas the temporal TNF-*α* release was initially low at 3 and 6 h and then presented a significant (*P* < 0.001) increase at 24, 48, and 96 h (Figure 9).

### 3.5. Overall Growth Factor and Cytokine Stability/Release from Hemocomponents

**Figure 10:**
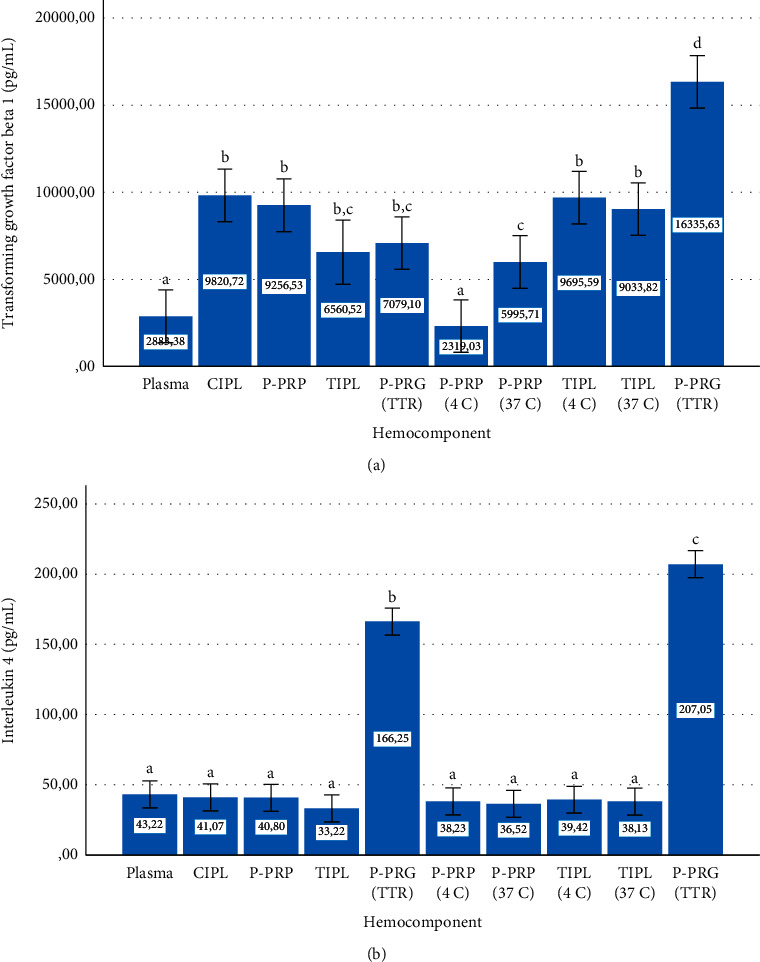
Overall mean ± sd transforming growth factor beta 1 (a) and interleukin 4 (b) concentrations either conserved or released from hemocomponents. ^a–c^Different lowercase letters denote significant (*P* < 0.001) differences between hemocomponents by the Games–Howell post hoc test.

**Figure 11:**
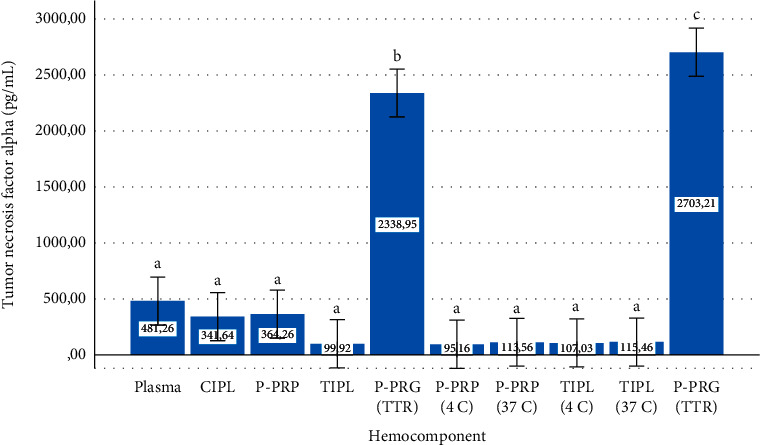
Overall mean ± sd tumor necrosis factor alpha either conserved or released from hemocomponents. ^a–c^Different lowercase letters denote significant (*P* < 0.001) differences between hemocomponents by the Games–Howell post hoc test.

General data analysis of the total concentration of each mediator is presented in Figures 10(a) and 10(b) and 11. Total TGF-*β*_1_ concentration was significantly (*P* < 0.001) lower in plasma and P-PRP stored at 4°C compared to CIPL, P-PRP (basal), TIPL, P-PRG (total temporal release), P-PRP stored at 37°C, and TIPL stored at both temperatures and P-PRPG total release (P-PRG (TR)).

Notably, P-PRG (total release) presented the significantly (*P* < 0.001) highest GF concentration in comparison with the total concentration of the same GF factor released by the rest of the hemocomponents (Figure 10(a)).

Total IL-4 (Figure 10(b)) and TNF-*α* (Figure 11) concentrations exhibited a similar stability/release pattern, which was characterized by the presence of significantly (*P* < 0.001) lower concentrations of both mediators in all hemocomponents, except P-PRG (total temporal release) and P-PRG (total release). Both mediators were significantly concentrated in the hemocomponents, with a significantly (*P* < 0.001) higher concentration in the P-PRG (total release) than in the P-PRG (total temporal release).

## 4. Discussion

The present study aimed to determine how the GF and cytokine release profiles from two bovine hemocomponents, P-PRP and TIPL, coming from a similar platelet concentrate source are directly affected by the method used, either chemical or physical, for stimulating the release of biomolecules and how the effects of temperature and time could affect the stability of these mediators in these hemocomponents.

In the present study, three key mediators, TGF-*β*_1_, IL-4, and TNF-*α*, were chosen to evaluate and compare the biochemical profile of P-PRP and TIPL. This selection included TGF-*β*_1_, which is a pleiotropic GF with important anabolic and anti-inflammatory actions that are mainly stored and released from PLT alpha granules, although it is also released in small quantities from WBCs [15–18]. IL-4 is an anti-inflammatory cytokine with anabolic properties in some tissues, such as cartilage [18–20]. This mediator is not directly released by PLT; however, its production is increased by the effect of cytokines contained in PRP that stimulate the target cells, such as the WBCs contained in the platelet-rich gel clots over time [21, 22]. On the other side, TNF-*α* is a cytokine with predominant proinflammatory and catabolic actions on cells and tissues [18, 23, 24].

As mentioned, one of the technical reasons why this research was performed was related to knowing what hemocomponent, either P-PRP or TIPL, could be more suitable for the treatment of dairy cows with clinical or subclinical mastitis [11, 13]. This aspect is of paramount importance because great volumes of homologous hemocomponents could be required for the clinical management of several cows on a dairy farm, bearing in mind that the incidence of this disease could vary between 19 and 52.7% [25]. On the other hand, it is probable that most dairy farms have limitations in producing their HCs because they could lack lab facilities or be located very distantly from a supplier of these biodrugs. Thus, from a theoretical point of view, the employment of ready-to-use frozen PLs as a treatment for any clinical form of mastitis [11] could be more technically suitable than using fresh PRP [13]. However, several intriguing results obtained in this research, which will be discussed in this section, could be useful for partially debunking the theoretical advantages of PL when compared to PRP.

The protocol used in this study allowed obtaining 1.7-fold platelet concentrations in relation to the initial concentration of these cytoplasmic fragments in whole blood, accompanied by an incipient concentration of WBCs, which is why these hemocomponents can be classified as P-PRP products [7].

The bioproduct evaluated in the present study is different from other bovine PRP preparations obtained by previously described double centrifugation protocols [9, 11], which allowed PRPs with high PLT counts to be obtained. On the other hand, information on the WBC concentration in these hemocomponents was only available for one study [9], which presented a very high concentration of these cells, indicating that this substance can be classified as an L-PRP. At this point, it is important to consider that the method used for producing platelet concentrates in the present study could have some technical advantages in comparison to the aforementioned protocols [9, 11] because great volumes of homologous hemocomponents can be obtained without broking the sterility barrier [26]. Furthermore, this protocol diminishes the work time and the use of sterile material, such as tubes, pipettes, and syringes, among other materials in comparison with the aforementioned protocols [9, 11].

Both positive (CIPL) and negative (plasma) controls were included in this study to determine the degree of biomolecule enrichment in the evaluated hemocomponents (P-PRP and TIPL) and establish the potential source (either cellular or plasma) of the mediators. Notably, Triton X 100 produced a massive release of TGF-*β*_1_ from platelet concentrates that were treated with this anionic detergent, which was considered CIPLs. Interestingly, the calcium-stimulated platelet concentrates (P-PRP) presented a very similar release of this mediator when compared to CIPLs. However, TGF-*β*_1_ release from TIPLs was approximately 40% less than that from CIPL and P-PRP. This situation could be associated with a deficient capacity of the frozen/thawing protocol to produce membrane cell damage in the same extension that when an anionic detergent is used [8]. On the other hand, basal IL-4 and TNF-*α* concentrations were similar between plasma, CIPL, TIPL, and P-PRP, which could indicate that these cytokines are scarcely stored in PLTs [18].

Several novel and intriguing findings were noticed when the effects of time and temperature were evaluated on the biomolecule concentrations in this study. TGF-*β*_1_ concentrations were drastically affected when P-PRPs were stored at 4 and 37°C over the first 3 h when compared to TIPLs stored at 4 and 37°C over 3 and 96 h and with the same P-PRP stored at 37°C at 96 h. Several explanations could be proposed to understand this finding, one of them is that possibly P-PRP at lower temperatures (4°C or cooler) loses the capability for producing additional TGF-*β*_1_ and consequently increases the consumption or denaturation of this GF. This assumption could be sustained by the fact that the same hemocomponent at 37°C was able to maintain statistically similar TGF-*β*_1_ concentrations when compared with TIPLs stored at both temperatures at any time. At this point, it is necessary to mention that scarce live cells (WBCs) contained in P-PRP at 37°C could initiate the novo synthesis of this GF and possibly avoid its denaturation.

On the other hand, although TIPLs presented higher TGF-*β*_1_ concentrations at 3 h, the same gradually diminished at 96 h. These GF concentrations were approximately 50% higher in TIPLs than in P-PRPs at 3 h. This finding could be explained by the fact that the membrane cell damage produced by the frozen/thawing protocol continued over time with an additional release of this GF after 3 h, accompanied by progressive denaturation of this biomolecule at 96 h. Overall, the results of this study related to TGF-*β*_1_ stability are novel and controversial because it is widely accepted that this GF is well stabilized at 4°C or cooler [18]. Regarding the effects of temperature and time on IL-4 and TNF-*α* concentrations in P-PRP and TIPLs, these biomolecules showed no changes in the concentrations of these hemocomponents stored at either 4 or 37°C. This finding is also contradictory since these cytokines are preferably stabilized under temperature conditions similar to those of TGF-*β*_1_ [18].

The biomolecule elution kinetics from P-PRGs performed in the present study were useful for demonstrating that P-PRP potentially remains an alive biomaterial over time (P-PRG), which produced a sustained release of GF and cytokines over time because it harbors cells into a 3D fibrin matrix, which induces the novo synthesis of GF and cytokines [27, 28]. On the other hand, it was observed that the P-PRP of this study maintained at 37°C formed a clot (P-PRG) 3 h after calcium gluconate activation. However, no clot evidence was observed for TIPL at any temperature or activated P-PRP at 4°C. These findings suggest that the ideal temperature for P-PRP activation and conservation over at least 96 h is 37°C. On the other hand, the overall results of this study could indicate that PRP could be more suitable for the treatment of cows with clinical forms of mastitis than frozen PLs.

P-PRGs under culture conditions, which could emulate an alive tissue or organ environment in which they are applied, actively produce mediators necessary to induce several biological reactions implicated in wound healing and inflammation [27, 28] and infection control [29], which could be of paramount importance for the potential treatment of cows with either clinical or subclinical mastitis.

## 5. Conclusions

The present study corroborates the initial hypothesis that platelet lysates produced by a physical process, in this case with extreme temperature variation (TIPLs), are hemocomponents different from P-PRP despite coming from the same bovine platelet concentrate. The elution mediator kinetics study demonstrated that TGF-*β*_1_ was produced by novo synthesis, which implies that those cells anchored in P-PRG clots are alive because they produce additional mediators over time. During the process of TIPLs, these hemocomponents are challenged with severe temperature changes that induce cell damage and release cells; thus, TIPLs lose the ability to anchor live cells and produce de novo synthesis of mediators. P-PRP activated with calcium gluconate and maintained at 37°C, which polymerizes in P-PRG, showed the best GF and cytokine release/denature profile compared with the rest of the hemocomponents evaluated.

## Data Availability

All data supporting the findings are included in the article. However, if readers need additional data for this study, these will be provided by the corresponding author, carmona@ucaldas.edu.co (J. U. Carmona).
